# Raman Spectroscopy Analysis for Optical Diagnosis of Oral Cancer Detection

**DOI:** 10.3390/jcm8091313

**Published:** 2019-08-27

**Authors:** Ming-Jer Jeng, Mukta Sharma, Lokesh Sharma, Ting-Yu Chao, Shiang-Fu Huang, Liann-Be Chang, Shih-Lin Wu, Lee Chow

**Affiliations:** 1 Department of Electronic Engineering, Chang Gung University, Taoyuan 333, Taiwan; 2 Department of Otolaryngology-Head and Neck Surgery, Chang Gung Memorial Hospital, Linkou 244, Taiwan; 3 AI Innovation Research Center, Chang Gung University, Taoyuan 333, Taiwan; 4 Department of Public Health, Chang Gung University, Taoyuan 333, Taiwan; 5 Green Technology Research Center, Chang Gung University, Taoyuan 333, Taiwan; 6 Department of Cardiology, Chang Gung Memorial Hospital, Taoyuan 333, Taiwan; 7 Department of Computer Science and Information Engineering, Chang Gung University, Taoyuan 333, Taiwan; 8 Department of Physics, University of Central Florida, Orlando, FL 32816, USA

**Keywords:** oral cancer, Raman spectroscopy, PCA-LDA, PCA-QDA, cryopreserved tissue

## Abstract

Raman spectroscopy (RS) is widely used as a non-invasive technique in screening for the diagnosis of oral cancer. The potential of this optical technique for several biomedical applications has been proved. This work studies the efficacy of RS in detecting oral cancer using sub-site-wise differentiation. A total of 80 samples (44 tumor and 36 normal) were cryopreserved from three different sub-sites: The tongue, the buccal mucosa, and the gingiva of the oral mucosa during surgery. Linear discriminant analysis (LDA) and quadratic discriminant analysis (QDA) were used with principal component analysis (PCA) to classify the samples and the classifications were validated by leave-one-out-cross-validation (LOOCV) and k-fold cross-validation methods. The normal and tumor tissues were differentiated under the PCA-LDA model with an accuracy of 81.25% (sensitivity: 77.27%, specificity: 86.11%). The PCA-QDA classifier model differentiated these tissues with an accuracy of 87.5% (sensitivity: 90.90%, specificity: 83.33%). The PCA-QDA classifier model outperformed the PCA-LDA-based classifier. The model studies revealed that protein, amino acid, and beta-carotene variations are the main biomolecular difference markers for detecting oral cancer.

## 1. Introduction

Oral cancer is one of the most common cancers globally and is the sixth most common malignancy, being closely associated with smoking, alcohol drinking, chewing tobacco, and consuming betel quid. The most common histology of oral cancer is squamous cell carcinoma (SCC) [1]. In males, it is the most common type of oral cancer and is found in various parts of the head and neck. It accounts for 90% of oral malignancies in 300,000 annually diagnosed cases [2]. According to Stewart [3], about 60% of new cases of oral cancer and 68% of deaths related to oral cancer reportedly occur in Asia. Oral squamous cell carcinoma (OSCC) is usually diagnosed late, resulting in an overall five-year survival rate of 50% [4]. The early detection and timely treatment of pre-malignancy may prevent oral potentially malignant disorders (OPMDs), which transform into oral cancer [5]. Although biopsies are the gold standard for diagnosing oral cancer, they are invasive and therefore painful, requiring an incision in the tissue. Since biopsies are time-consuming and invasive, clinicians increasingly favor non-invasive techniques such as vital staining, light-based detection, and other optical diagnostic technologies [6].

Raman spectroscopy (RS) is an optical technique that is both fast and simple. It is one of the most widely-used techniques for the non-destructive characterization of molecules and materials [7]. RS probes the vibrational modes of a molecule, which are sensitive to its chemical bonds and provides a unique “fingerprint” that enable the identification of chemicals [8]. Most non-invasive techniques, such as light-based detection and optical diagnostic techniques, have great potential for screening and monitoring OPMDs [6]. However, no standalone method can accurately identify OPMDs. RS is a rapidly emerging technique with medical applications in the early diagnosis of various types of cancer [9,10,11]. Schut et al. [12] studied the effectiveness of RS for the in vivo classification of normal and dysplastic tissue by measuring the palatal tissues of rats. Sundar et al. [13] studied the application of RS to oral tissues in both normal and malignant stages. Changes in the ratio of relative intensities can be useful in analyzing oral tissues to detect oral malignancy. Many studies have demonstrated the use of RS to distinguish between normal and malignant, or among normal, pre-malignant, and malignant forms of oral mucosa using various preservation techniques and analytical methods [14,15,16,17,18,19,20]. In Lau et al. [21] studied the use of RS to differentiate normal from cancerous nasopharyngeal tissues. Lau [22] differentiated three stages of cancers in larynx tissue. Some authors have used exfoliated [23] and serum-based samples [24,25] to differentiate between normal and abnormal tissues. RS also has the potential to provide an objective intra-operative evaluation of the cancer surgical margins, favoring the detection of any residual tumor after surgery [26,27,28].

In this investigation, cryopreservation with fresh excision was used to study the efficacy of RS for classifying normal and tumor tissues. Cryopreservation is a process that preserves organelles, cells, tissues, and any other biological structures. In this work, samples of tissues were extracted after surgery and immediately preserved in liquid nitrogen. This method helped to prevent the alteration of the structure and morphology of tissue. Linear discriminant analysis (LDA) and quadratic discriminant analysis (QDA) classifiers with principal component analysis (PCA) were used to distinguish tumor from the healthy tissue structures of oral mucosa. Sub-site-wise differentiation of tongue cancer versus normal, buccal cancer versus normal, and gingiva cancer versus normal were performed. Generally, the QDA model is a better classifier than the LDA model. However, in some cases, the LDA model is better than the QDA model. Therefore, both the LDA and QDA models were studied. This work is the first to attempt the sub-site-wise differentiation of cryopreserved tissue samples using RS.

## 2. Materials and Methods

### 2.1. Patients and Samples

This study was approved by the Institutional Review Board (IRB) of Chang Gung Medical Foundation (IRB No: 201800420B0), Taiwan. The study was conducted in the Department of Otolaryngology Head and Neck Surgery with the written and informed consent of the enrolled participants. All specimen and pathological reports were collected at Chang Gung Memorial Hospital for analysis. A total of 36 normal and 44 tumor cryopreserved tissue samples with histologically-proven malignancies and normal oral mucosa were collected, as shown in Table 1. The sub-sites were identified and recorded at the time of surgery and specimen acquisition. Normal tissues were taken from a site adjacent to the tumor. The study period was from February 2017 to December 2018. A total of 80 tissue samples were collected (from the tongue, buccal mucosa, and gingiva), which included 36 normal and 36 tumor samples from the same patients and eight tumor samples from different patients. All samples were at least 3×3 mm in size. Surgical resection specimens from normally appearing mucosa adjacent to the tumor were taken 15–30 min following surgical excision whereas the tumor samples were obtained immediately after surgery. The distance from tumor border to the adjacent tissue or resection margin was 1.5 to 2 cm. The tested cryopreserved samples were freshly cut and kept in liquid nitrogen (N${}_{2}$) at −80 °C to prevent alteration to its morphology until its use. Each tissue was analyzed by RS. Glass was used as the substrate to test each tissue sample because it is the most widely available substrate and yields good results at lower source wavelength [29]. A total of 44 samples of tumor cells and 36 normal cells were obtained. Five spectra of each tissue sample were obtained at different locations. Since five spectra were obtained from each tissue sample, 220 and 180 spectra of tumor and normal tissues were respectively obtained, yielding 400 spectra for analysis by point-wise approach and 80 by patient-wise approach (five spectra per tissue averaged to yield one spectrum: 80 = 400/5).

### 2.2. Pre-Processing and Data Analysis

Data were processed and analyzed in MATLAB (R2018a, MathWorks, USA). First, a Savitsky–Golay filter (with order = 3) was used to smooth the recorded spectra so as to remove interference. After baseline correction, the area under the curve (AUC) technique was used to normalize all spectra to eliminate the data redundancy. The AUC is a function in MATLAB software. It normalizes a group of spectra with peaks by standardizing the area under the curve to the group median. Each value in a sample is divided by the sum over the sample. Unsupervised PCA was applied to the normalized spectrum from 700 to 2000 cm${}^{-1}$. Normalized spectra were fed to multivariate the supervised classifier models PCA-LDA and PCA-QDA.

The LDA and QDA classifier models were used to study the boundary between classes and probabilities of classification. These models maximize the ratio of “between class variance” to “within class variance”. This results in diminishing the data variation in the same class and detachment between classes. The LDA classifier assumes a common co-variance matrix and generates a linear boundary, while the QDA classifier assumes that each class has its own co-variance and produces a quadratic boundary. QDA optimally discriminates between the classes in the dataset [30], and requires large computation and data. Therefore, LDA is a good classifier for equal class samples and QDA is a good classifier for unequal class samples [31]. However, in some cases, they perform worse than expected [32]. To evaluate the classify results, the classifier models were optimized using a training dataset and their performance was evaluated using a test dataset.

### 2.3. Raman Spectroscopy (RS)

A RS instrument (ProTrusTech Co., Ltd., Taiwan) that comprised of a laser with a wavelength of 532 nm as an excitation source and a laser power of 126 mW was used. Spectral acquisition proceeded as follows. The excitation wavelength was 532 nm, the laser power was 6.3 mW∼12.6 mW, the integration time was 5 s, the acquisition time was 15 s, and the average value of spectrum was 3 (meaning that the display spectrum averaged from three scanning spectra). The spectra resolution, specified by the manufacturer, was 1 cm${}^{-1}$. The laser spot size was 6∼8 micron and the penetration depth was 10∼20 micron.

### 2.4. Multivariate Analysis

The mean normalized spectrum was analyzed using two supervised classifier models, PCA-LDA and PCA-QDA. PCA is a statistical procedure that reduces the number of dimensions and provides principal components (co-ordinates) based on new dimensions. The number of PCA components was less than half of the minimal sample classes to avoid over-fitting [25]. The first three principal components (PC1, PC2, and PC3) accounted for up to 97% variance, as evaluated by PCA. They were fed into the LDA and QDA classifiers. For PCA-LDA and PCA-QDA, scores of factor 1, 2, and 3 were chosen to obtain a three-dimensional scatter plot with a decision boundary. The analysis broadly categorized normal and tumor tissues under point-wise and patient-wise methods. In the point-wise approach, five spectra of a sample were analyzed. Owing to the heterogeneity of the tissue, the measured spectra at the various point varied greatly in intensity and Raman shift. In the patient-wise approach, one average spectrum of each sample was analyzed to eliminate heterogeneity.

## 3. Results and Discussion

A total of 80 tissue samples from three sub-sites were analyzed. The data were to be distinguished in the following two-class systems: Normal versus tumor, tongue tumor versus normal, buccal mucosa tumor versus normal, and gingiva tumor versus normal. The spectral features, vibrational molecules, and analysis of the three sub-sites will be described below.

### 3.1. Finger Print Region

**Figure 1:**
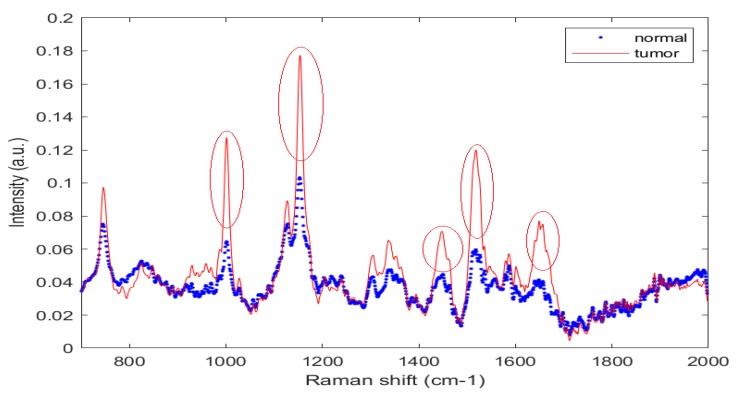
Mean spectra of oral normal and tumor cryopreserved tissues.

Figure 1 presents normalized mean spectra of healthy or normal and tumor or OSCC tissues of the oral mucosa. The fingerprint region (700 cm${}^{-1}$ to 1800 cm${}^{-1}$) in biological tissues is rich in proteins, nucleic acids, amino acid, carbohydrates, and lipids. The literature has shown that the normal tissue spectral peaks are lipid-dominated peaks while the malignant tissue peaks are protein-dominated peaks [14,15,19,33,34]. Malignant or tumor samples had higher peaks than normal tissues. Peaks at 1004, 1156, 1339, 1450, 1523, and 1656 cm${}^{-1}$ dominated the spectra of normal tissues, whereas peaks at 754, 1064, 1168, and 1220 cm${}^{-1}$ dominated those of malignant or tumor tissue samples. The sharp and high peak at 1004 cm${}^{-1}$ is attributed to the symmetric ring breathing mode of phenylalanine, which is an amino acid and is observed in protein-enriched malignant tissue spectra [14]. A sharp and intense peak at 1155∼56 cm${}^{-1}$ arose from the proteins and was dominated by the protein signal in the tumor tissues [27,34]. The peak at 1220∼1240 cm${}^{-1}$ is associated with lipids and =CH bending. The high peak at 1449∼50 cm${}^{-1}$ is associated with CH${}_{2}$ bending, and is associated with a protein [33,34]. The peaks at 1339 cm${}^{-1}$ in the average tumor spectrum was associated with the adenine feature of nucleic acid [23]. At 1518∼1524 cm${}^{-1}$, a sharp and more intense peak is observed and is associated with the beta-carotene or porphyrin feature and was obtained in both normal and tumor samples [35]. The lower intensity from normal tissues may be a discharge by secretion. In one study [36], this peak was absent from the spectra of normal tissues. A broad and strong peak at 1650∼1655 cm${}^{-1}$ is a characteristic of proteins in the alpha-helix structure of amide I, which yields a strong signal in the spectral of tumor tissues. In normal tissues, the small peak at 1655 cm${}^{-1}$ is generated by the C=C bond in lipids or phospholipids, and not amide I [14,34]. Normal tissues yielded a small peak at 1123 cm${}^{-1}$, which is attributable to the C−C skeletal stretch in lipids, while tumor tissues had a (C−N) stretching mode of protein. Tumor tissues yielded a high peak at 750 cm${}^{-1}$ and a small peak at 823 cm${}^{-1}$ due to the Tryptophan and Tyrosine in protein, respectively [35]. Normal tissues yielded Raman peaks at 754, 1064, 1168, and 1220–84 cm${}^{-1}$ (=CH bending) that are associated with the lipid. All of the above peaks were obtained from both normal and tumor tissues, with the strongest signals at 750, 1004, 1155, 1449, 1522, and 1656 cm${}^{-1}$. In tumor tissue samples, protein, amide I, greater CH${}_{2}$ bending, amide III, and amino acid (Tryptophan or phenylalanine) yielded signals that enabled such tissue to be distinguished from normal tissues.

**Figure 2:**
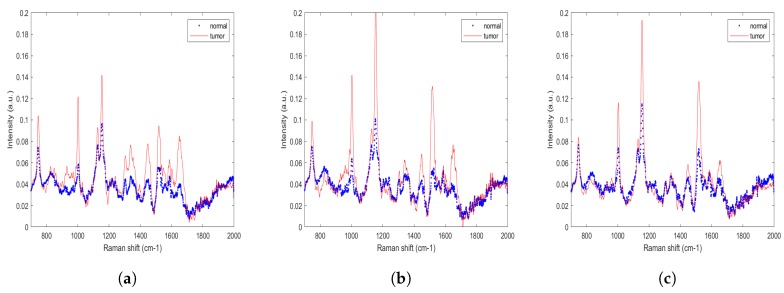
Mean spectra of: (**a**) Buccal mucosa, (**b**) gingiva, and (**c**) tongue.

Figure 2a–c present the normalized mean Raman spectra of tumor and normal samples from three sub-sites (buccal, gingiva, and tongue). Tumor/normal samples at all sub-sites yielded almost identical mean Raman spectra, but the three sub-sites did not yield the same intensity in corresponding regions. According to one study [37], different sub-site of oral mucosa (tongue, buccal, gingiva, hard palate, and soft palate) have different percentages of collagen and elastin. Carvalho et al. [38] demonstrated the biochemistry associated with healthy oral tissues at each sub-site and differentiated them using the basis of specific biochemical components. Figure 2 reveals that amide I and amide III bands at 1655 and 1250 cm${}^{-1}$, respectively, were more prominent at the buccal tumor sub-site than at the gingiva and tongue tumor sub-sites. Protein/lipids bands at 1155 and 1523 cm${}^{-1}$ were more intense at the tongue and gingival sub-site than at the buccal sub-site. All three sub-sites were known to vary with respect to prognosis, metastasis to lymph nodes, aggressiveness, and overall survival rate. These different genetic alterations and biological differences will be responsible for the basis of classification among buccal, tongue, and gingiva cancer [39,40,41].

### 3.2. Analysis of Normal and Tumor Sample Data

The confusion tables for PCA-LDA and PCA-QDA classifiers were generated. The performance parameters were calculated from the confusion tables (correct and incorrect predictions). Point-wise and patient-wise approaches were analyzed using PCA-LDA and PCA-QDA classification models. To evaluate their performance, their accuracy, sensitivity, and specificity in identifying normal and tumor tissues were calculated. All of the spectra were subjected to PCA. Three PCA components were used for classification using both LDA and QDA models.

**Figure 3:**
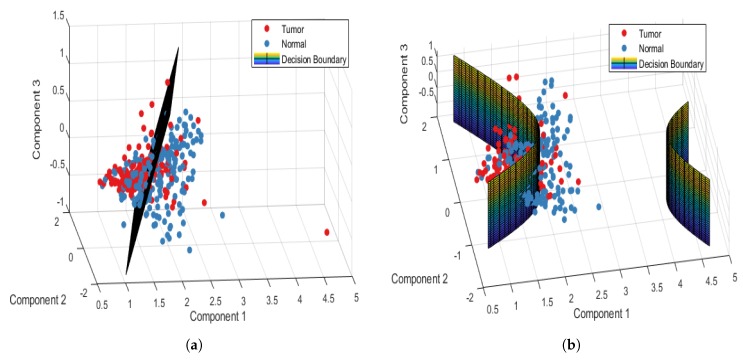
Point-wise 3D decision boundary curve for (**a**) PCA-LDA and (**b**) PCA- QDA classifier model.

**Figure 4:**
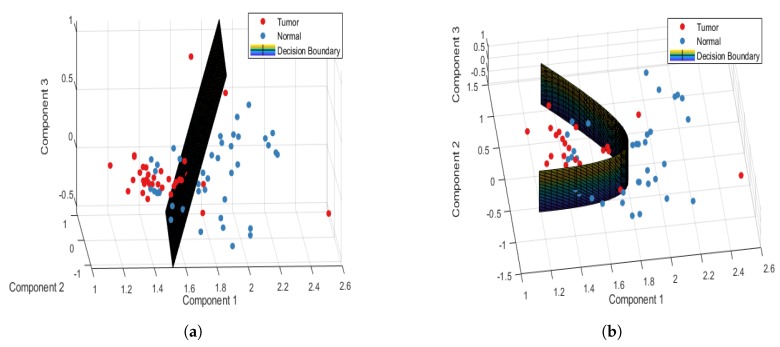
Patient-wise 3D decision boundary curve for (**a**) PCA-LDA and (**b**) PCA-QDA classifier model.

Table 2 shows the confusion and performance tables of normal and tumor sample data, analyzed using the PCA-LDA and PCA-QDA model for the point-wise approach. The PCA-LDA model correctly classified 177/220 and 121/180 tumor and normal sample data, respectively. However, the PCA-QDA model correctly classified 184/220 and 143/180 tumor and normal sample data, respectively. The accuracy, sensitivity, and specificity of the PCA-LDA model in differentiating normal and tumor tissues were 74.5%, 80.45%, and 67.22%, respectively. However, the accuracy, sensitivity, and specificity of the PCA-QDA model in distinguishing normal and tumor tissues were 81.75%, 83.63%, and 79.44%, respectively. Figure 3a,b plot the 3D decision boundary curves for normal and tumor sample data using point-wise approach for the PCA-LDA and the PCA-QDA classifier model, respectively. The decision boundary classified the tumor and normal sample data. The solid red and blue dots represent tumor and normal classes, respectively. Table 3 shows the confusion and performance tables of normal and tumor sample data, analyzed using the PCA-LDA and PCA-QDA model for the patient-wise approach. The PCA-LDA model correctly classified 34/44 and 31/36 tumor and normal sample data, respectively. The PCA-QDA model correctly classified 40/44 and 30/36 tumor and normal sample data, respectively. The accuracy, sensitivity, and specificity of the PCA-LDA model in differentiating normal and tumor tissues were 81.25%, 77.27%, and 86.11%, respectively and those of PCA-QDA model were 87.5%, 90.90%, and 83.33%, respectively. The PCA-QDA model therefore exhibited a better classification performance than the PCA-LDA model. Figure 4a,b plot the 3D decision boundary curve of the patient-wise approach for the PCA-LDA and the PCA-QDA classifier model, respectively. The patient-wise approach is seen as a better classifier than the point-wise approach. Therefore, only the patient-wise approach was used in the following three sub-site analyses.

### 3.3. Analysis of Data from Normal and Tumor Samples from Tongue, Buccal Mucosa, and Gingiva Sub-Sites

**Figure 5:**
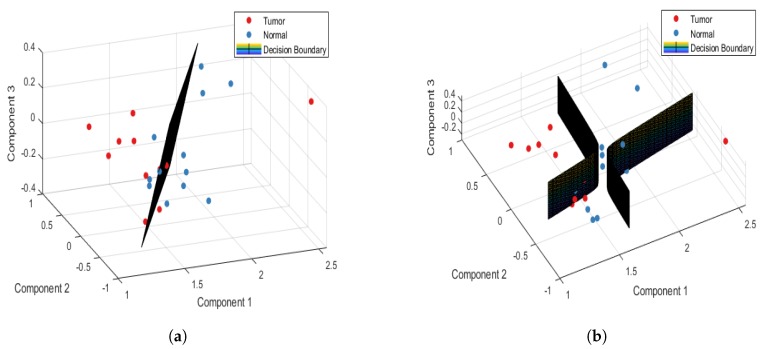
Tongue patient-wise 3D decision boundary curve for (**a**) PCA-LDA and (**b**) PCA-QDA classifier model.

Spectra of 13 tumor samples and 11 normal samples at the tongue sub-site were collected. Table 4 shows the performance table of the patient-wise approach at the tongue sub-site. The PCA-LDA model differentiates tumor and normal tissues had an accuracy, sensitivity, and specificity of 79.16%, 92.3%, and 63.63%, respectively, and the PCA-QDA model did so with corresponding values of 87.5%, 100%, and 72.72%, respectively. Figure 5a,b plot the 3D decision boundary curve of the PCA-LDA and PCA-QDA classifier models with the tongue sample data, respectively. In Table 4, specificity quantifies the extent to which persons without a disease undesirably screen as positive. The accuracy (1-error rate) is the proportion of correct predictions, including correct positive and negative predications based on the selected samples [42]. The low specificity of the tongue indicates miss-classification between healthy tissue sublayers (surface squamous epithelium, muscle, and gland) and the OSCC structure, which has been discussed in an earlier study [43] and the section on validation methods.

**Figure 6:**
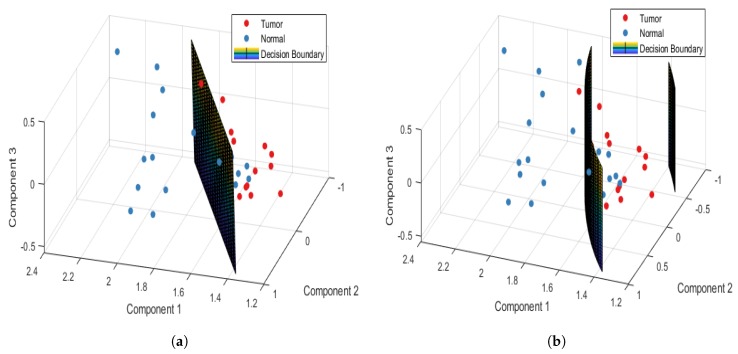
Buccal mucosa patient-wise 3D decision boundary curve for (**a**) PCA-LDA and (**b**) PCA-QDA classifier model.

At the buccal mucosa sub-site, 19 tumor samples and 14 normal samples were collected. Table 5 shows the performance table of the patient-wise approach at the buccal mucosa sub-site. The PCA-LDA model differentiates the buccal mucosa tumor and normal tissues with an accuracy, sensitivity, and specificity of 84.84%, 78.94%, and 92.85%, respectively, and the PCA-QDA model differentiates them with 87.87%, 84.21%, and 92.85%, respectively. Figure 6a,b plot the 3D decision boundary curve of the PCA-LDA and PCA-QDA classifier models, respectively with buccal mucosa sample data. For the PCA-QDA model, the increasing sensitivity results with increasing accuracy are because the true positive cases are more correctly identified in this model. The PCA-QDA model outperformed the PCA-LDA model.

**Figure 7:**
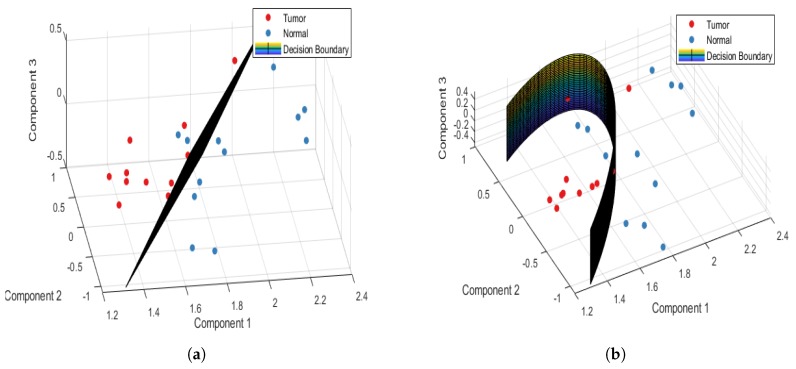
Gingiva patient-wise 3D decision boundary curve for (**a**) PCA-LDA and (**b**) PCA-QDA classifier model.

From the gingiva sub-site, 12 tumor samples and 11 normal samples were collected. Table 6 shows the performance table of the patient-wise approach at the gingiva sub-site. The PCA-LDA model differentiates gingiva tumor from normal tissue with an accuracy, sensitivity, and specificity of 91.30%, 91.66% and 90.90%, respectively, and the PCA-QDA model differentiates them with 87.12%, 75%, and 100%, respectively. Figure 7a,b plot the 3D decision boundary curve of the PCA-LDA and the PCA-QDA classifier models with the gingiva sample, respectively. The PCA-LDA model assumed linearity and variance-covariance homogeneity, whereas the PCA-QDA model had different feature covariance matrices for different classes and the consistency of a PCA-QDA model could not be predicted from a few samples, reducing the accuracy of the PCA-QDA model.

## 4. Validation Methods

Cross-validation is a process by which the performance of a model is estimated using a limited number of data sample. It estimates the effectiveness of machine learning models (PCA-LDA and PCA-QDA models herein) with unseen data. The sample dataset is randomly partitioned into two disjoint subsets (training and validation data sets). The validation dataset is used to evaluate the performance of the model [44]. The cross-validation methods herein were k-fold and leave-one-out-cross-validation (LOOCV). In the k-fold method, the parameter K is the number of groups. A given dataset is randomly split into K equal subsets. Each subset is called as a fold and is unique. One fold was used as the test data set and the other K-1 folds were used as a training data set. The machine learning classification model was trained using the training data set and the performance parameters were evaluated. This process was iterated for the K folds and the performance parameters were aggregated. In the LOOCV method, a single sample within a given dataset was used as the test data and the other samples within a given dataset were used as training data. This process was iterated until each sample in a given dataset was used once as the test data. Therefore, each sample held out from the training data set. This method requires a large computation time because many iterations are required for training. The LOOCV method aggregates the estimated error rate by the number of samples in a given dataset. The k-fold method repeatedly randomizes a sub-sampling that can be used for training and testing data set in all the samples. However, the LOOCV method does not require a random process. Hence, in the LOOCV method, the estimation has less in bias but high variance [45]. However, in the k-fold method, the reduction of variance increases the value of K. Therefore, the bias remains low.

Table 7 shows estimates of the error rates of the model for point-wise and patient-wise approaches. The error rates of the PCA-LDA and PCA-QDA models for the point-wise approach were 25.5% and 16.27%, respectively. However, the error rates of the PCA-LDA and PCA-QDA models for patient-wise approach were only 18.75% and 12.5%, respectively. These results were confirmed by the k-fold and LOOCV methods, which yielded error rates of 18.25% and 17% for the point-wise approach, and 16.25% and 11.25% for the patient-wise approach, respectively. These results support the conclusion that the patient-wise approach is better than the point-wise approach. Therefore, only the patient wise approach was used in the following validation.

Table 8 provides estimates of the models’ error rates at the tongue, buccal mucosa, and gingiva sub-sites. At the tongue sub-sites, both PCA-LDA and PCA-QDA models had low specificity because the validation methods generated higher error rates than other sub-sites. Since the oral tissue is heterogeneous, it comprises of different structures and layers. Cals [43] reported that the sub-layers of a healthy tissue structure (surface squamous epithelium, muscle) have the same protein, lipids, and nucleic acid as OSCC or tumor tissues of the tongue. Therefore, the miss-classification between healthy tissues sublayers (surface squamous epithelium, muscle, and gland) and the OSCC structures was greater than other sub-sites. In earlier studies [27,28], the same miss-classification observed between OSCC and surface squamous epithelium was observed owing to the low specificity of the classification model when applied to tongue tissues. The PCA-LDA and PCA-QDA models reveal that most of the biomolecular information from tissues and cells are critical in discriminating tumorous tissues from healthy tissues. This diagnostic model can also differentiate subgroups using the different components of Raman biochemical and biomolecular features and thus sub-site oral cancer can be distinguished from normal tissue.

The limitations of this work include the limited number of samples at each sub-site (tongue: 24, buccal mucosa: 33, and gingiva: 23). Our future investigations will target a maximum number of samples for each sub-site in the oral cavity to enhance the classification rate and use other approaches that involve meta-learning and neural networks for classification.

## 5. Conclusions

This work studied the application of RS to oral cryopreserved freshly-excised tissue samples. This method had several advantages, including its rapidity, lack of need for labeling, and inexpensiveness. It has the potential to improve the efficiency of screening procedures for oral cancers and to identify the boundary for tumor-free resection margin during surgery. The PCA-QDA model for the patient-wise approach had greater classification efficiency than the PCA-LDA model. In the future, we will develop artificial intelligence algorithms to classify data and reduce the error rate.

## Figures and Tables

**Table 1:** Description of all tested cryopreserved tissue samples under Raman spectroscopy.

Sub-Sites	Tongue	Buccal Mucosa	Gingiva	Total
Tumor	13	19	12	44
Normal	11	14	11	36

**Table 2:** Confusion and performance tables for point-wise approach.

Dataset	Confusion Table			Performance Parameters		
**PCA-LDA**	Tumor	Normal	Total	Accuracy (%)	Sensitivity (%)	Specificity (%)
Tumor	177	43	220	74.50	80.45	67.22
Normal	59	121	180			
**PCA-QDA**	Tumor	Normal	Total	Accuracy (%)	Sensitivity (%)	Specificity (%)
Tumor	184	36	220	81.75	83.63	79.44
Normal	37	143	180			

**Table 3:** Confusion and performance tables for patient-wise approach.

Dataset	Confusion Table			Performance Parameters		
**PCA-LDA**	Tumor	Normal	Total	Accuracy (%)	Sensitivity (%)	Specificity (%)
Tumor	34	10	44	81.25	77.27	86.11
Normal	5	31	36			
**PCA-QDA**	Tumor	Normal	Total	Accuracy (%)	Sensitivity (%)	Specificity (%)
Tumor	40	4	44	87.50	90.90	83.33
Normal	6	30	36			

**Table 4:** Performance table of patient-wise tongue analysis.

Patient-Wise: Tongue	Accuracy (%)	Sensitivity (%)	Specificity (%)
PCA-LDA	79.16	92.30	63.63
PCA-QDA	87.50	100.00	72.72

**Table 5:** Performance table of patient-wise buccal mucosa analysis.

Patient-Wise: Buccal	Accuracy (%)	Sensitivity (%)	Specificity (%)
PCA-LDA	84.84	78.94	92.85
PCA-QDA	87.87	84.21	92.85

**Table 6:** Performance table of patient-wise gingiva analysis.

Patient-Wise: Gingiva	Accuracy (%)	Sensitivity (%)	Specificity (%)
PCA-LDA	91.30	91.66	90.90
PCA-QDA	87.12	75.00	100.00

**Table 7:** Error Rate of PCA-LDA, PCA-QDA, and validation methods for normal versus tumor.

Error Rate	PCA-LDA (%)	PCA-QDA (%)	Validation: K-fold (%)	Validation: LOOCV (%)
Point-wise	25.50	16.27	18.25	17.00
Patient-wise	18.75	12.50	16.25	11.25

**Table 8:** Error Rate of PCA-LDA, PCA-QDA, and validation methods for each sub-site.

Error Rate	PCA-LDA (%)	PCA-QDA (%)	Validation: K-fold (%)	Validation: LOOCV (%)
Tongue	20.83	12.50	16.67	16.67
Buccal	15.16	12.13	18.18	21.21
Gingiva	8.60	12.88	13.04	19.04
